# Cold temperatures, stress, and violence

**DOI:** 10.1016/j.heliyon.2019.e01619

**Published:** 2019-05-14

**Authors:** Pavel N. Prudkov, Olga N. Rodina

**Affiliations:** a Ecomon ltd., Russian Federation, Yaroslavskoe shosse 4-1-60, Moscow, 129337, Russia; b Department of Psychology, Moscow State University, Mohovaja 8-5, Moscow, 103009, Russia

**Keywords:** Clinical psychology, Epidemiology, Public health, Psychiatry, Psychology

## Abstract

The relation between temperature and violence was found in many studies. However, the results of such studies demonstrated only that uncomfortably hot temperatures increase violence. There seem to be no data on the effect of cold temperatures. We studied the relation between temperature and violence for the Russian Federation because the Russian Federation is a country with huge climatic differences. Two types of the analysis of the data were applied. In Analysis 1 average yearly temperatures were used. For violent crimes a decrease in temperature resulted in the increase of the crimes after taking into account three socioeconomic variables. Analysis 2 was based on monthly data. Violence was high in winter and spring months but low in autumn months. In our opinion, the conventional models that are used to clarify the effect of hot temperatures cannot explain our results. We hypothesize that long periods of cold temperatures can be considered as mild chronic stress. Chronic stress may exert depression and depression is associated with irritability and anger. In some situations these emotions may stimulate violence. An increase in violence associated with city living and economic downturns may partially be a consequence of mild chronic stress.

## 1 Introduction

The relation between temperature and aggression was already noticed in the Ancient Greece and Rome (Anderson et al., 2000). However, until recently there have been not adequate statistical approaches to this problem. Modern studies with appropriate statistical techniques started about 60 years ago and became especially intensive nowadays (Anderson, 1987, 2001, Burke et al., 2015; Simister and Cooper, 2005). The results of these studies can be formulated as the temperature-aggression hypothesis that suggests uncomfortable temperatures increase aggressive actions (Anderson et al., 2000).

The temperature-aggression hypothesis, in principle, can be separated in two assumptions. The heat hypothesis assumes that uncomfortably hot temperatures cause increases in aggression. The heat effect is the empirical demonstration of an increase in aggression and violence in uncomfortably hot temperatures. The cold hypothesis states increases in aggression may result from uncomfortably cold temperatures. Accordingly, the cold effect is the empirical demonstration of an increase in aggression and violence due to cold (Anderson et al., 2000).

Most of studies are associated with the validation of the heat hypothesis and data on the cold effect are “quite rare in the literature” (Anderson et al., 2000, p. 65). As a result, it may be assumed that there exists no cold effect (Van Lange et al, 2017). However, the cold effect may be important for understanding mechanisms underlying the relation between temperature and aggression. If the cold effect occurs then there is a range of temperatures in which the influence of temperature on aggressive motivation and behavior is minimal. This means that the functional relation between temperature and aggression can be presented as the U-function. The lack of the cold effect corresponds to a straight linear function or the J-function. The J-function shows the nonlinear but monotonic relation between an increase in temperature and aggression.

Although, at present there is still no unequivocal explanation for the relation between temperature and aggression two theories seem to be popular. One of them is the General Aggression Model (Anderson and Bushman, 2002). This model assumes that ambient temperature directly and rapidly influences feelings, thoughts and the physiology of the organism. Uncomfortable temperatures make people angry, increase aggressive thoughts and blood pressure. As a result, the threshold of aggressive actions is decreased and in provocative situations this may result in aggression and violence. As there may be a range of comfortable temperatures, the General Aggression model expects the relation between temperature and aggression may correspond to the U-function.

The Routine Activity Theory (Cohen and Felson, 1979; Rotton and Cohn, 2001) suggests another explanation for the relation between temperature and violence. The basic idea of the Routine Activity Theory is very simple – during the warmer periods of time individuals are more likely to leave their homes, schools, and jobs, and spend more time in outdoor public spaces, where contacts with other individuals can become aggressive. The Routine Activity Theory expects that the relation between temperature and aggression can be presented as the J-function.

The cold effect may be, hence, a useful method to validate these popular models. In our opinion, one of the reasons why the data on the cold effect is still missing is that practically all studies are based on the data from countries with relatively or very warm climates such as the USA (Anderson, 1987; Simister and Cooper, 2005) and India (Mishra, 2015; Simister, 2008). However, there exist quite a number of countries with cold climates and the Russian Federation is one of them. The Russian Federation is a huge country which consists of 83 regions (federal subjects) with very large climatic differences. The most southern regions are situated at the latitude of Rome and Madrid but the country also includes the republic of Sakha (Yakutia) which is the coldest place in the Northern Hemisphere (“Yakutia”, n. d.). The population of 81 regions exceeds 100,000 (Oxenoit et al., 2015). This means that all statistics can be considered representative. Moreover, the population of the Russian Federation is relatively homogenous, the ethnic group of Russians comprises 81 percent of the population and Russians predominate in most of the regions ("Russia", n. d.). This means that cultural and social differences between the Russian regions are relatively narrow. We decided to study the relation between temperature and violence using data on the regions of the Russian Federation.

There was a particular characteristic of the available data: the data on crimes and social factors were available for the entire regions only and unavailable for the locations within the regions (its capitals, other cities, etc). On the contrary, the data on temperatures were available for capitals and unavailable for entire regions. As a result, we used the data on crimes from entire regions and the data on temperatures from its capitals. We consider that this approach did not influence our results significantly. It is reasonable to assume that the temperatures in the capital of a region strongly correlate with the temperatures in the entire region. Moreover, in all regions the capital is the most populated city (Oxenoit et al., 2015). A median capital concentrates 37.4 percent of the population of the region. Hereafter, when we mention temperatures in a region we really mean temperatures in its capital.

## 2 Analysis 1

### 2.1 Methods

Analysis 1 was based on annual data. The crime rates (i.e. the number of crimes per 100000 people) for each region were calculated for all of available types of crimes: murder + assault, rape, larceny, drug trafficking, tax evasion. The data on these crimes were borrowed from (Ulianov et al., 2006; Oxenoit et al., 2015).

Socioeconomic factors can obviously affect the relation between temperature and violence. We used three socioeconomic variables to control the relation. One of them was average regional income per capita. Some findings have demonstrated that there is an association between incomes per capita and crimes (Coccia, 2017; Van de Vliert, 2013). The second variable was regional life expectancy. The ability to evaluate one's life expectancy (Griskevicius et al., 2011) can influence individual's behavior. Shorter life expectancies are associated with greater willingness to engage in aggression and violent criminal acts, whereas the opposite relations are found for longer life expectancies (Dunkel and Mathes, 2011). This association is especially important for Russia because Russia is known for a short life expectancy with average 70.93 years in 2014 (Oxenoit et al., 2015). Since the vast majority of crimes are committed by males, in our analysis we used regional male life expectancy. The data on average income and male life expectancy were found in (Oxenoit et al., 2015).

As many of violent crimes are influenced and accompanied by alcohol use disorder it should be mentioned that alcohol abuse is a very serious problem for the Russian Federation. According to the World Health Organization, in 2010 alcohol consumption per capita in the Russian Federation was 15.1 equivalent liters of pure ethanol (World Health Organization, 2014) which was the fourth position in the world. Alcoholism was the third socioeconomic variable. We used regional ratings of alcoholism from (“National rating of sobriety”, n. d.). A rating of alcoholism is an integrative indicator which is based on such parameters as the number of deaths caused by alcoholism, vodka and beer sales. This rating was significantly correlated with the murder + assault rate in 2014 (r = 0.55, p = 0.001).

Other socioeconomic factors (income inequality, urbanization, etc) may influence crime. However, income per capita and life expectancy are usually considered main socioeconomic variables that determine social and economic processes in an area and other socioeconomic factors are correlated with these two variables frequently (Van de Vliert, 2013). Therefore, we suggest that the two basic variables plus alcoholism which influences crime in the Russian Federation seriously are the minimally sufficient number of variables to evaluate the effect of socioeconomic factors on crime in the country.

Because there may be significant correlations between average annual temperature and socioeconomic variables this can result in effects of multicollinearity in regression analyses such as the unreliability of the p-values of regression coefficients (Cohen et al., 2013). To reduce such effects we used ridge regression for all analyses below (Cohen et al., 2013). The variance inflation factor (VIF) is usually considered a characteristic of multicollinearity. If the VIF calculated for an independent variable is more than five then the multicollinearity of the variable is high (O'Brien, 2007). All VIFs computed in our analyses were less than five.

The data on average annual temperatures in the capitals of the regions were found at www.pogodaiklimat.ru and www.meteoinfo.ru.

### 2.2 Results and discussion

To define the relation between temperature and violence we calculated the coefficients of correlation between average annual temperature and crime rates. It is obvious that climatic conditions and crime rates may fluctuate between years, therefore correlations for one year cannot be considered sufficiently representative. As a result, we computed these coefficients for two years. 2014 was selected because the data on alcoholism were available only for this year. The data on 2004 were used to demonstrate the effect under consideration was stable within 10 years. The coefficients of correlations between average annual temperature and five crime rates for 2004 and 2014 are presented in Table 1.

**Fig. 1:**
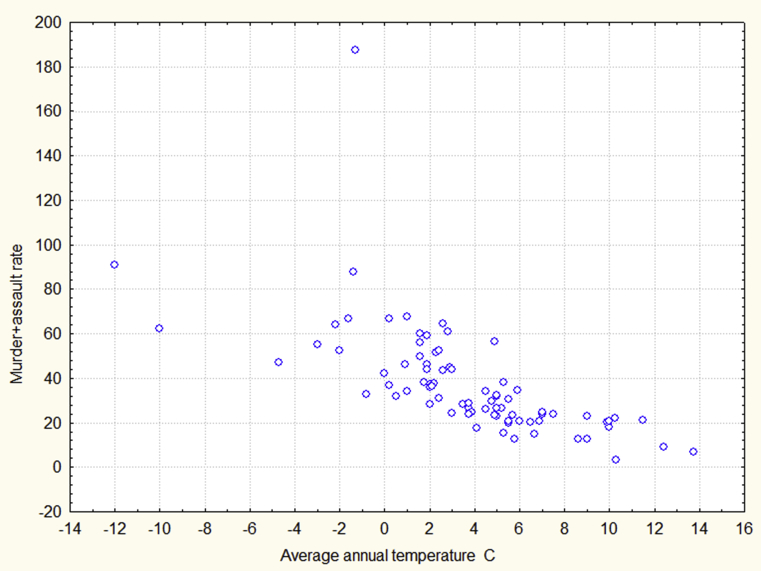
Average annual temperature versus the murder + assault rate in 2014.

Table 1 shows four from the five crime rates are negatively and significantly correlated with average annual temperature for one year at least and the correlations between temperature and the murder + assault rate are especially strong and evident. The relation between annual average temperature and the murder + assault rate for 2014 is presented in Fig. 1.

**Table 1:** Correlations between average annual temperature and crime rates.

Year	2004					2014				
Average crime rate	Murder + assault 61.18	Rape 6.04	Larceny 873.12	Drug trafficking 96.9	Tax evasion 154.89	Murder + assault 30.76	Rape 2.84	Larceny 609.62	Drug trafficking 167.88	Tax evasion 48.43
Average annual temperature	-0,53	-0,27	-0,41	-0,21	-0,20	-0,66	-0,36	-0,44	-0,28	0,14

Significant (p < 0.05) coefficients are underscored.

It is of interest to mention that the decrease in crime which occurred from 2004 to 2014 results in more significant correlations. The decrease can probably be explained by the improvement in social conditions caused by the economical upturn in the Russian Federation for this period. The Russian Gross Domestic Product increased by 38.45 percent from 2004 to 2014 (“Sdelano u nas”, n. d.). In other words, the deterioration of social and economic conditions underlying crime leads to more significant correlations. This fact, likely, means that, as a result of such deterioration, the role of unfavorable climate tends to increase; therefore, climatic conditions may really influence crime.

However, the results from Table 1 are insufficient to confirm the cold effect because social factors are not taken into consideration here. Average annual temperature for the 83 regions significantly (p < 0.001) correlated with average income (r = -0.49), male life expectancy (r = 0.61), and alcoholism (r = 0.64) and these variables can influence criminal actions more than average annual temperature. All correlations and other calculations henceforth were carried out for 2014, because as was points out above the regional alcoholism ratings were available for this year only.

To control socioeconomic factors we applied multiple regression analysis. Regression coefficients were calculated for the four significant crime rates. In each analysis one of these crime rates was a dependent variable and average annual temperature, average income, male life expectancy, and alcoholism were independent variables. The region with the highest violent rate in Fig. 1 (the Tyva Republic) was considered an outlier and excluded from all analyses. The beta coefficients are presented in Table 2.

**Table 2:** Beta coefficients for the four dependent variables.

Dependent variables Independent variables	Murder + assault	Rape	Larceny	Drug trafficking
Average annual temperature	-0.462482***	-0.281753*	-0.073433	-0.050284
Average income	0.057601	-0.209209	0.044733	0.210546
Male life expectancy	-0.327145^∗∗^	-0.063337	-0.492907^∗∗∗^	-0.220118
Alcoholism	0.070963	0.204028	0.033069	-0.061015

* - p < 0.05; ** - p < 0.01; *** -p<0.001.

Table 2 demonstrates that cold temperatures significantly influence the murder + assault rate even after taking socioeconomic factors into account which means that the cold effect for these crimes does exist. There is the cold effect for rape crimes although it is weak. Table 2 shows that temperature does not affect larceny and drug trafficking.

Looking at Fig. 1 one may say that our data are distinctly different from the data obtained in other studies and therefore the causes of our data may be special and nonstandard and as a consequence, our results cannot be used for the understanding of general mechanisms which define links between temperature and violence. Indeed, Fig. 1 seems only to demonstrate the cold effect: as temperature rises violence tends to decrease even for relatively high temperatures (higher than 10 degrees Celsius). However, other studies show that for such temperatures the heat effect can already be detected (Simister, 2008).

It is not difficult to notice that Fig. 1 can be separated in two parts. For low temperatures (lower than 3–4°) the slope of the graph is steep, for higher temperatures the slope becomes more horizontal. This possibly implies that the relation between temperature and violence for low and high temperatures can be determined by different mechanisms. To examine this assumption we decided to separate the whole sample in cold and warm regions and repeat the regression analyses for both groups. Because there is no clear criterion for separation we, first, computed a mean temperature for the whole sample (3.71°) and then separated all regions in 39 cold regions (where average annual temperatures are not higher than 3.71°) and 43 warm regions (where average annual temperatures are higher than 3.71°) and we computed regression analyses for the cold and warm regions separately. The beta coefficients for the dependent variable murder + assault are presented in Table 3.

**Table 3:** The beta coefficients for the cold and warm regions.

Murder + assault Independent variables	The cold regions	The warm regions
Average annual temperature	-0.398634**	-0.027659
Average income	0.109220	0. 73449
Male life expectancy	-0.588234^∗∗∗^	-0.426924^∗^
Alcoholism	-0.057782	0.311203

* - p < 0.05; ** - p < 0.01; *** -p<0.001.

Table 3 shows the cold effect occurs in the cold regions only. А decrease in average annual temperature by 1 degree Celsius resulted in an increase in the murder + assault rate by 2.62 in 2014. For the warm regions the effect of temperature disappears. Our data for relatively high temperatures are, hence, consistent with other studies. It is important to note there is no effect of temperature on the other crimes in the cold regions.

## 3 Analysis 2

Analysis 1 we have demonstrated the cold effect: as temperature decreases the murder + assault rate increases in the cold regions of the Russian Federation. A skeptic may object that because Analysis 1 was based on average annual temperatures the cold effect obtained in Analysis 1 is possibly a consequence of some socioeconomic or cultural variables that affect crimes in the cold regions only. Moreover, the correlations between the dependent variables mean that the results of the regression analyses in Analysis 1 are not absolutely reliable. To demonstrate the existence of a real cold effect it is necessary to show that during the periods of cold temperatures, for example, during winter months the murder + assault rate is higher than during other seasons. Socioeconomic and cultural variables cannot influence such a result. The purpose of Analysis 2 was to consider the relation between ambient temperature and crime on a monthly basis.

### 3.1 Methods

The monthly data on murder + assault rates for 83 regions through 2010–2015 years were found at www.crimestat.ru. Thus, there were 498 observations for each month. Table 1 demonstrates that murders and assaults were being decreased during these years; therefore, for these crimes we used the relative monthly rate (henceforth, RMR-MA) for each month of each indicated year and each region in our analysis. RMR-MA was calculated as follows:

(1) $$\text{RMR}-\text{MA}\;=\frac{\left(365\;\text{or}\;366\right)*\left(\text{monthly}\;\text{murders}+\text{assaults}\right)\;}{\left(\text{the}\;\text{number}\;\text{of}\;\text{days}\;\text{in}\;\text{the}\;\text{month}\right)*\left(\text{yearly}\;\text{total}\;\text{murders}+\text{assaults}\right)}$$

If the RMR-MA for a month in a year exceeds 1 this means that in an average day of this month people committed murders and assaults more frequently than in an average day of this year. There were calculated 498 RMR-MAs for each month.

In Analysis 1 it was demonstrated that after taking socioeconomic variables into account cold temperatures affect murders and assaults but does not affect nonviolent crimes such as larceny. It was of interest to study how temperatures influence the monthly rates of larceny but such data were unavailable. Instead, the monthly data on all crimes were used. Because murders and assaults made only 2.4% of all crimes in 2014 (Oxenoit et al., 2015), it is reasonable to suggest that the data on all crimes allow to demonstrate how temperatures affect nonviolent crimes. The relative monthly rate for all crimes (henceforth, RMR-C) was calculated as follows:

(2) $$\text{RMR}-\text{C}\;=\;\frac{\left(365\;\text{or}\;366\right)*\left(\text{monthly}\;\text{crimes}\right)\;}{\left(\text{the}\;\text{number}\;\text{of}\;\text{days}\;\text{in}\;\text{the}\;\text{month}\right)*\left(\text{yearly}\;\text{total}\;\text{crimes}\right)}$$

498 RMR-Cs were calculated for each month.

Average monthly temperatures for 67 capitals were available. 402 observations for each month were used. These data were found at www.pogodaiklimat.ru.

### 3.2 Results and discussion

**Fig. 2:**
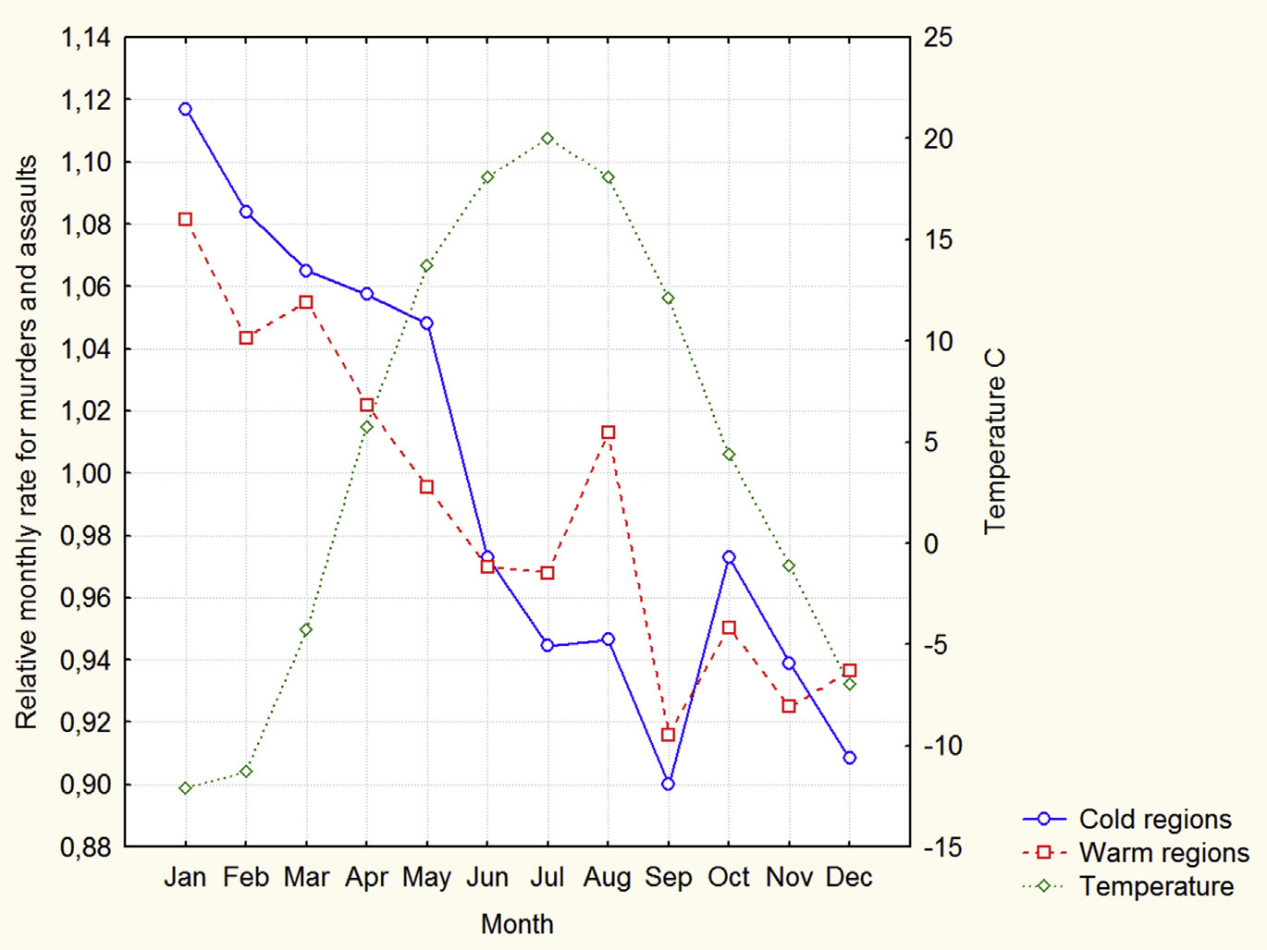
Monthly distributions of medians for murder + assault and temperature.

The monthly distributions of the median for the relational rates for murders + assaults and the monthly distribution of the median for temperatures are presented in Fig. 2.

**Fig. 3:**
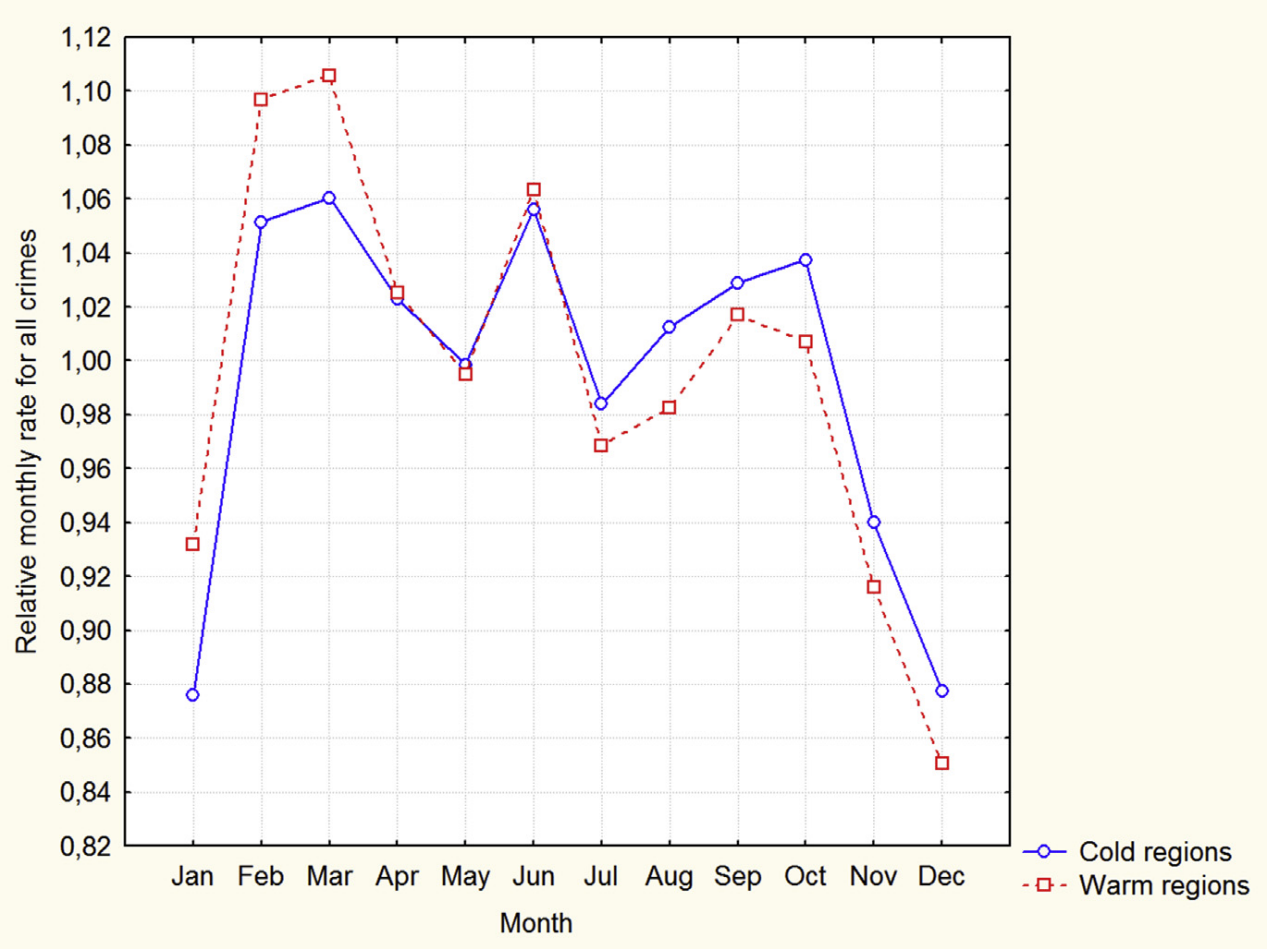
Monthly distributions of medians for all crimes.

Fig. 2 demonstrates that maximal RMR-MAs are in winter and spring months in both cold and warm regions. Unlike the symmetrical distribution of temperatures, both distributions of RMR-MAs are strongly asymmetrical. The monthly distributions of the median for the relational rates for all crimes are presented in Fig. 3.

These distributions seem seasonally and regionally uniform. The correlation coefficients between average monthly temperatures and RMR-MAs are presented in Table 4.

**Table 4:** Correlations between average monthly temperatures and relative monthly violence.

	Month											
	Jan	Feb	Mar	Apr	May	Jun	Jul	Aug	Sep	Oct	Nov	Dec
Cold regions	.08	.04	-.19	.05	-.02	.07	-.02	.05	.07	.08	-.03	-.03
Warm regions	.04	0.01	.11	.02	-.16	.06	.02	.17	.01	-.20	.01	.12

Significant (p < 0.05) coefficients are underscored.

**Fig. 4:**
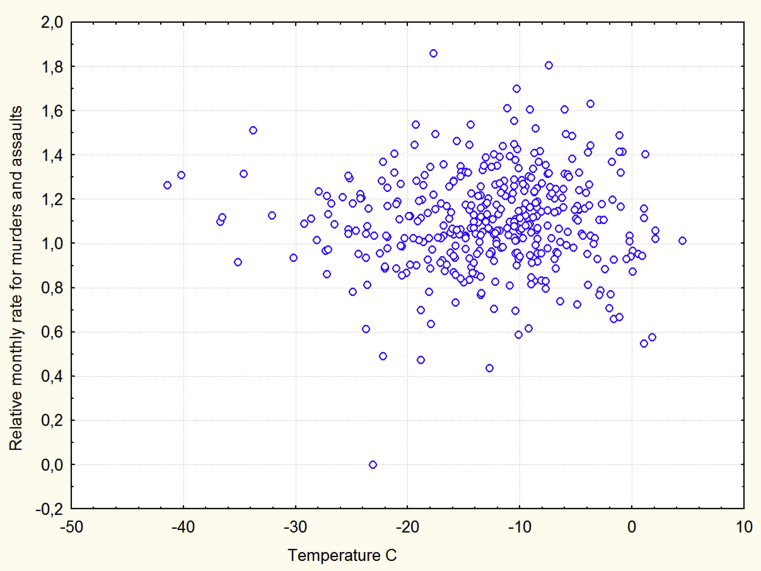
Temperature versus violence in January.

Table 4 shows that the correlations between average temperatures and violence are, in general, low and insignificant. The association between monthly violence and average monthly temperature for all available regions in January is presented in Fig. 4.

In our opinion, Fig. 2 clearly demonstrates the cold effect, which is more striking in the cold regions. Moreover, Fig. 2 shows that violence does not follow ambient temperatures because violence is higher at the end of winter and in spring than in autumn and at the beginning of winter in all regions, although, for example, the temperature in spring months is approximately the same as that in autumn months.

There are weak correlations between monthly violence and average monthly temperature; however, correlation coefficients are sometimes insufficient to understand the association between two variables. The graphical presentation of the variables may be useful. Fig. 4 presents the association between temperature and violence for all available data in January. Fig. 4 clearly shows that huge (about fifty degrees Celsius) differences in average monthly temperatures between the observations do not result in changes in violence.

Fig. 2 demonstrates a local peak of violence in the warm regions in August and there is a significant positive correlation coefficient between temperature and violent crimes in these regions in this month. These results probably correspond to the heat effect. Fig. 3 shows that unlike the distributions for the murder + assault rate, there are no clear seasonal and/or regional effects in the distributions for all crimes. The heat effect only for the most violent crimes is similar to the results of other studies (Anderson et al., 2000). This is another piece of evidence that the results of our research are not unique and can be applied to elucidate the general mechanisms underlying the relation between temperature and violence.

## 4 Discussion

The influence of temperature on aggression and violence has been discussed since Antiquity. However the use of adequate statistical techniques for exploration of this phenomenon has started relatively recently. The results of numerous studies demonstrate the existence of the heat effect, i.e., the increase of violence at uncomfortably hot temperatures. The increase of violence in uncomfortably cold temperatures, i.e., the cold effect is theoretically possible; however, the data demonstrating it seem to be missing. The cold effect can be useful for understanding hypothetical mechanisms underlying the relation between temperature and violence. To find the cold effect we decided to study links between temperature and violence in the Russian Federation because the Russian Federation has very large climatic varieties from region to region.

In Analysis 1 that is based on yearly data it is demonstrated that there is a cold effect in some regions of the Russian Federation because in these regions with cold climates murder + assault rates are higher than in the regions with warmer climates after taking into consideration three socio-economic variables which correlate with ambient temperature.

However, these results seem insufficient to show the cold effect because if yearly data are used then some regional factors rather than temperature itself may determine violence. Analysis 2 is based on the monthly data for six years from 2010 to 2015. The results of Analysi 2 clearly demonstrate higher murder + assault rates in winter and spring. Moreover, in these seasons the violence in the cold regions is higher than that in the warm regions.

One may argue that our data are not sufficient to demonstrate the cold effect reliably because there is some discrepancy between the source of the data on temperature and the source of the data on crime. Moreover, average monthly temperatures are not available for all capitals. As is pointed out above, we do not suggest that the discrepancy affects our results seriously. Also, the main results of Analysis 2, that is, the cold effect and the asymmetry in the distribution of violence within the year are independent from specific ambient temperatures because in all places in the Northern Hemisphere winter months are the coldest season and the distribution of temperatures within the year is symmetrical.

If the cold effect is evident in the Russian Federation this raises a question why the cold effect is so rare in other findings. It is reasonable to assume that simply the Russian Federation is a very cold country. For example, Norway and Iceland are usually considered the countries with cold climates. However, the average annual temperature in Oslo, the capital of Norway is 5.69 degrees Celsius and that in Reykjavík, the capital of Iceland is 4.3 (“Oslo”, n. d; “Reykjavík”, n. d.). In other words, in the Russian Federation these cities would be considered as the warm regions.

The heat effect in the warm regions in August which is consistent with the predictions of the conventional models of the temperature –aggression relation (the General Aggression Model and the Routine Activity Theory), means these models, to some extent, explain the temperature-aggression relation in the Russian Federation. However, the conventional models are not sufficient to explain most of the results obtained in our study. The conventional models assume that ambient temperature causes violence directly and instantaneously. Because the distribution of temperatures within the year is symmetrical, the conventional models predict the symmetrical distribution of violent crimes. However, Fig. 2 clearly demonstrates that these distributions are strongly asymmetrical. The conventional models also expect rather strong correlations between average monthly temperatures and monthly violence, however, these correlations are, in general, weak. As was pointed out above, the most southern regions of the Russian Federation are situated at the latitudes of Italy and Spain but some other regions are among the coldest in the world. Therefore, the difference in cold temperatures among the regions must be maximal in January because January is the coldest month. If cold temperatures directly cause violence then in January the difference in violence between cold and warm areas also must be maximal. However, Fig. 4 shows that this is not the case. It is important to note that any model that assumes that temperature or a factor associated with temperature directly and instantaneously exerts violent actions may face similar problems.

Humans evolved in hot climates of Africa (Klein, 2009), therefore, they seem to consider very cold climates unsuitable for living. For example, the population of Alaska, which is the coldest state of the United States, is only 0.23 percent of the total population, although the area of Alaska makes 17.4 percent of the total area of the US (“Alaska”, n. d.) The area of the regions of the Russian Federation with average annual temperature not higher than 0° is 44.06 percent of the total area, but its population was only 5.37 percent of the total population in 2014 (Oxenoit et al., 2015). There may be various reasons why people avoid living in very cold climates such as the difficulty of farming and very high costs of the construction and maintenance of infrastructure at extremely low temperatures, but it is reasonable to assume that the climate itself plays a major role.

We hypothesize the long periods of extremely cold temperatures along with the shortage of daylight hours during winter months may exert depressive moods in many people. Depression is frequently associated with irritability and anger (Winkler et al., 2005; Painuly et al., 2005). In provocative situations irritability and anger can stimulate aggressive actions and violence. Unlike the conventional models, our hypothesis does not suggest that the cold ambient temperature directly affects feelings and thoughts. Instead, aggressive motivations are results of the accumulation of depressive moods caused by living in extremely cold temperatures. This effect may be especially strong if average annual temperatures are lower than 3–4 degrees Celsius because at higher average temperatures the duration of the periods of low temperatures are probably not sufficient to exert continuous depressive moods in people.

We have not the exact data on the depressive moods in the Russian Federation, however, migration can be considered the proxy for people's moods. Obviously, people tend to leave some places if they are not satisfied with their life there and positive emotions of the residents of other areas may stimulate some people to migrate to such places. There was a significant correlation (r = 0.3847, p = 0.000) between average annual temperature and the migration rate in 2014. The positive correlation coefficient implies that people leave regions with cold climates and relocate to warm climates. Ambient temperature possibly is not a major factor that stimulates people to migrate. Most probably people are guided by socioeconomic conditions. However, after taking average income and male life expectancy into account the partial correlation between average annual temperature and migration rate stays significant (0.251, t (79) = 2.31, p = 0.023). The data on migration, hence, are consistent with the assumption that the residents of regions with cold climates have considerable depressive moods.

Seasonal affective disorder (SAD) is a combination of biologic and mood disturbances (Rohan et al., 2009). SAD usually occurs in autumn and winter months with a decrease in spring and summer seasons. Community-based studies demonstrate that the prevalence of SAD approaches 10 percent in the northern latitudes (Byrne and Brainard, 2008). Patients with SAD have some symptoms of depression. Therefore, one may suggest that the hypothetical depressive mood underlying violence is the seasonal affective disorder in reality. SAD is associated with the shortage of daylight rather than cold temperatures. To define the role of the duration of daylight hours we found the latitudes of the capitals for all cold regions because the seasonal changes in the length of the daylight hours are determined by local latitudes. We repeated the regression analysis for the cold regions with latitude as an additional independent variable. The regression coefficient for latitude was insignificant and the addition of latitude did not affect the significance of the other independent variables. It seems that our hypothetical depressive moods cannot be reduced to SAD.

The hypothesis that violence is probably a consequence of the accumulation of depressive moods may clarify the results obtained in Analysis 2. Because the accumulation is a slow process this explains why the cold effect does not occur in November and December and starts only in January. The depreciation of depression is also a slow process especially in the cold regions, therefore, violence may be higher in these regions in spring months than in autumn months. The accumulation hypothesis, hence, explains the asymmetry in the distributions of violence within the year. The accumulation hypothesis does not assume that temperature exerts violent actions instantaneously and this is consistent with weak correlations between monthly temperatures and monthly violence. The accumulation hypothesis, in a similar vein, explains why there is a weak association between temperature and violence in January. In our opinion, irritability and anger caused by the accumulation of depression during long periods of cold temperatures may be a main cause of the cold effect in the Russian Federation. Of course, further studies are necessary to clarify the role of various variables in the cold effect in more detail.

Long periods of extremely cold temperatures accompanied by the shortage of daylight hours can be considered as chronic stress. Numerous studies find that chronic stress exerts impairments in mental health such as depression and burnout (Marin et al., 2011). Chronic stress is also associated with post-traumatic stress disorder (Schneiderman et al., 2005). However, to our knowledge there are no findings which demonstrate chronic stress may directly exert aggression and violence. On the other hand, the assumption that chronic stress may stimulate violence through depression which, in its turn, results in irritability and anger is logically admissible.

There may be several reasons why the effect of chronic stress on violence has been not found so far. One of these reasons is that this effect seems to be relatively weak and, therefore, it can be detected only if many people are exposed to chronic stress for long periods of time. Additionally, stress is very frequently a consequence of violence (Schneiderman et al., 2005). When people are exposed to violence they often intentionally respond to violence by means of other violence simply because this may be the most effective survival strategy. As a result, it is difficult to detect and separate all sources of violence under such conditions. We suggest our results, which are based on the data obtained from millions of people exposed to chronic stress not being a consequence of violence, may be considered a confirmation of the increase in aggression and violence caused by mild, chronic stress.

The hypothesis that mild chronic stress may stimulate aggression needs further research. Some studies demonstrate that crime rates in urban territories are higher than in rural areas (Bailey, 1984; Ladbrook, 1988). Some researchers show that city living is associated with mental problems such as mood and anxiety disorders (Peen et al., 2010; Lederbogen et al., 2011). Therefore, city living may be considered a source of chronic stress and depression. If after evaluating urban and rural types of depression and controlling socioeconomic variables the contribution of depression to the difference between rural and urban crime rates remains significant, this may show the evidence favoring the association between mild chronic stress and violence.

Table 1 demonstrates that the improvement of the socioeconomic situation in a country conditioned by economic upturn results in the reduction of violence. Accordingly, the worsening of social conditions caused by economic downturns may raise violence. For example, the murder rate in the Russian Federation increased from 10.4 per 100000 people in 1990 to 21.3 in 1995 (Ulianov et al., 2006). Whereas, GDP was shrank by 38 percent (“Sdelano u nas”, n. d.) during this period of time. Negative social factors (poverty, unemployment, inequality) can stimulate some people to act more violently because in the situation of the impairment of social conditions the benefits of violent actions and crimes can exceed its costs. On the other hand, these negative factors can exert chronic stress. The assumption that mild chronic stress caused by social impairments increases violence can be examined by studying the relation between the negative social factors, depressive moods and violence in various situations associated with economic downturns and upturns.

## 5 Conclusions

We conducted two analyses regarding the relation between temperature and violence for the Russian Federation because the Russian Federation is a country with cold climates and huge climatic differences. In Analysis 1 which was based on the annual data for five types of crime and ambient temperature in 2004 and 2014 we found that only for murder + assault a decrease in temperature resulted in the increase of the crimes in the coldest regions after taking into account three socioeconomic variables. The monthly data on murder + assault rate and temperature through 2010–2015 years were used in Analysis 2. The results of Analysis 2 show that maximal murder + assault rates were in winter and spring. In our opinion, our research clearly demonstrates that very cold temperatures exert violence. Analysis 2 shows the discrepancy between the asymmetrical distribution of violence and the symmetrical distribution of temperature within the year. This result is inconsistent with the conventional models of the relation between temperature and violence. We assume the cold effect in the Russian Federation is a consequence of mild chronic stress and depression caused by living in extremely cold temperatures. An increase in violence associated with city living and economic downturns may partially be a consequence of mild chronic stress.

## Declarations

### Author contribution statement

Pavel N. Prudkov: Conceived and designed the experiments; Performed the experiments; Analyzed and interpreted the data; Wrote the paper.

Olga N. Rodina: conceived and designed the experiments; Performed the experiments; Contributed reagents, materials, analysis tools or data.

### Funding statement

This work was supported by the Russian Foundation for Fundamental Studies [scientific project number 17-06-00994].

### Competing interest statement

The authors declare no conflict of interest.

### Additional information

No additional information is available for this paper.
